# Neurorehabilitation using the virtual reality based Rehabilitation Gaming System: methodology, design, psychometrics, usability and validation

**DOI:** 10.1186/1743-0003-7-48

**Published:** 2010-09-22

**Authors:** Mónica S Cameirão, Sergi Bermúdez i Badia, Esther Duarte Oller, Paul FMJ Verschure

**Affiliations:** 1 Laboratory of Synthetic Perceptive Emotive and Cognitive Systems (SPECS), Department of Technology, Universitat Pompeu Fabra, Edifici la Nau, Roc Boronat 138, 08018 Barcelona, Spain; 2 Servei de Medicina Física i Rehabilitació, Hospital de L'Esperança, Barcelona, Spain; 3 Institució Catalana de Recerca i Estudis Avançats (ICREA), Barcelona, Spain

## Abstract

**Background:**

Stroke is a frequent cause of adult disability that can lead to enduring impairments. However, given the life-long plasticity of the brain one could assume that recovery could be facilitated by the harnessing of mechanisms underlying neuronal reorganization. Currently it is not clear how this reorganization can be mobilized. Novel technology based neurorehabilitation techniques hold promise to address this issue. Here we describe a Virtual Reality (VR) based system, the Rehabilitation Gaming System (RGS) that is based on a number of hypotheses on the neuronal mechanisms underlying recovery, the structure of training and the role of individualization. We investigate the psychometrics of the RGS in stroke patients and healthy controls.

**Methods:**

We describe the key components of the RGS and the psychometrics of one rehabilitation scenario called Spheroids. We performed trials with 21 acute/subacute stroke patients and 20 healthy controls to study the effect of the training parameters on task performance. This allowed us to develop a Personalized Training Module (PTM) for online adjustment of task difficulty. In addition, we studied task transfer between physical and virtual environments. Finally, we assessed the usability and acceptance of the RGS as a rehabilitation tool.

**Results:**

We show that the PTM implemented in RGS allows us to effectively adjust the difficulty and the parameters of the task to the user by capturing specific features of the movements of the arms. The results reported here also show a consistent transfer of movement kinematics between physical and virtual tasks. Moreover, our usability assessment shows that the RGS is highly accepted by stroke patients as a rehabilitation tool.

**Conclusions:**

We introduce a novel VR based paradigm for neurorehabilitation, RGS, which combines specific rehabilitative principles with a psychometric evaluation to provide a personalized and automated training. Our results show that the RGS effectively adjusts to the individual features of the user, allowing for an unsupervised deployment of individualized rehabilitation protocols.

## Background

Stroke is one of the main causes of adult disability [1] and of burden of disease in high- and middle-income countries with about 16 million first event stroke incidents per year [2-4]. Hence, both the economical and the psycho-social impact of stroke emphasize that we need to find effective diagnostics, treatment and rehabilitation approaches.

Recovery after a stroke relies on neuronal plasticity that allows other areas of the brain to take over functions of the ischemic zone, the complexity of this reorganization strongly depends on the severity of the anatomical and functional lesion [5-7]. Therefore, the main target of rehabilitation after stroke should be to maximize the effect of plasticity and functional reorganization. Several methods and therapy concepts have been proposed and many of them aim at promoting functional changes within surviving motor networks [8-15]. However, it is not always clear how effective these different approaches are and how they exactly influence recovery.

Relatively novel tools in neurorehabilitation are based on Virtual Reality (VR) technologies, these have the advantage of flexibly deploying scenarios that can be directed towards specific needs. Several VR systems have been proposed for the rehabilitation of motor deficits following stroke with particular emphasis on the rehabilitation of the upper limb and the hand (see [16-18] for reviews). Although a significant amount of work has been done in this area with promising results, the relevant characteristics of these systems and the quantification of their impact on recovery are not yet clearly understood [18]. As a result, we do not know how the different parameters of the proposed VR scenarios exactly affect recovery or whether they are effective at all. Furthermore, there is a need to take into account individual variability in the deficits and the behavior of the subjects in order to optimize the impact of training [19].

To address and investigate these aspects we have developed the Rehabilitation Gaming System (RGS), a VR based neurorehabilitation paradigm for the treatment of motor deficits resulting from lesions to the central nervous system that exploits the cognitive processes that mediate between perception and action [20,21]. RGS combines individualization with a brain based training rationale. In the following paragraphs, we describe the main considerations related to the design of this system.

The RGS tracks arm and finger movements and maps them onto a virtual environment. In this manner, the user controls the movements of two virtual limbs that are viewed in a first person perspective. The rehabilitation scenario described here, Spheroids, consists of intercepting, capturing and placing spheres that move towards the user. The main rationale behind this rehabilitation scenario of RGS is the hypothesis that bimanual task oriented action execution combined with the observation of virtual limbs that mirror the executed or intended movement create conditions that facilitate the functional reorganization of the motor and pre-motor systems affected by stroke. In the action execution and observation paradigm, recovery could be promoted through the engagement of undamaged primary or secondary motor areas or by recruiting alternative perilesional or contralesional networks. This, however, requires that an information channel must exist that allows external modulation of the states of these alternative circuits. We hypothesize that such an interface could be provided by neurons such as those found in the mirror neuron system, which have the property of being active both during the execution of goal-oriented actions with a biological effector and during the observation of the same actions performed by biological effectors of other agents [22-26]. It is exactly this cognitive transduction channel between the perception and execution of action that RGS exploits even when motor actions themselves cannot be performed due to a lesion. Indeed, recent studies have suggested a benefit of using passive action observation for rehabilitation following stroke [13].

In the mirror neuron literature, the perceptual frame of reference is often not considered and the mirror neurons are mainly reported in a third person perspective. However, it has been acknowledged that these neurons essentially follow the statistics of the multi-modal inputs the acting brain is exposed to [24]. This is consistent with current theories of perceptual learning that emphasize the role of sampling statistics in the development of perceptual structures [27,28]. For instance, it has been proposed that through statistical inference, associating motor intention and actions, the mirror neurons facilitate the encoding of the intentions of others [29]. Based on these observations, RGS assumes that the first person view should provide the most effective drive onto these multi-modal populations of neurons simply because this is the perspective that the system is most frequently exposed to. Indeed, it has been observed that the first person view of a virtual representation of the hand induces stronger activation of primary and secondary motor areas associated with sensory motor control as opposed to only performing hand movements in the absence of such a representation [30]. More concretely, the response is stronger when the orientation of the hand is similar to the one of the first person perceiver [31,32].

Since the Yerkes-Dodson law established the relationship between motivation and learning, it has been acknowledged that human performance is optimal at intermediate levels of arousal [33,34]. This means that the optimum experience in any task is the one that is perfectly balanced so as to be neither too hard nor too easy [35]. Given these considerations individualization means to identify a level of performance, i.e. failure rates, that optimally challenge each user at their own level of competence. Hence, any automated therapy system should be able to assess the performance level of the subject and subsequently tune the therapeutic intervention in relation to this level. Therefore, we quantitatively assessed the effect of each game parameter of the Spheroids training scenario on the task performance of stroke patients and healthy controls. This data was used to define a multi-dimensional psychometric model of the Spheroids RGS training scenario that could support a Personalized Training Module (PTM) that automatically adjusts the difficulty of the task with respect to the measured performance of a subject.

Finally, RGS, as any other VR based rehabilitation approach, assumes that training in virtual environments will lead to corresponding improvements in performance in the physical world. Therefore, to understand the transfer of performance between the virtual and the physical world, stroke patients and controls performed physical and virtual versions of a calibration reaching task. We show that individual movement properties and deficits are consistently transferred between real and virtual worlds, supporting the equivalence of training and acting in both environments.

Our results indicate that by virtue of the above properties, the Rehabilitation Gaming System is a promising neurorehabilitation tool that can be used to alleviate the deficits brought on by lesions to the central nervous system as the ones caused by stroke.

## Methods

### Participants

For the development of the Personalized Training Module (PTM), 10 control subjects (8 males and 2 females, mean age 29.0 ± 6.1 years) and 12 hemiplegic patients (11 males and 1 female, mean age 57.4 ± 12.1 years, 126.8 ± 108.2 days after stroke) participated in the trials. For the assessment of the PTM and the study of transfer between physical and virtual tasks two new groups of controls and patients were enrolled. 10 control subjects (8 males and 2 females, mean age 28.6 ± 3.6 years) and 9 patients (4 males and 5 females, mean age 62.3 ± 11.7 years, 13.1 ± 4.9 days after stroke) participated in the study.

The control subjects were students with no history of neurological disorders recruited from the SPECS Laboratory at the Universitat Pompeu Fabra in Barcelona. All patients were receiving rehabilitation at the Hospital de L'Esperança in Barcelona (see Table 1 for details). Patients were required to pass the Mini-Mental State Examination with a minimum score of 22 (over 30) [36]. We excluded patients that displayed emotional and/or cognitive deficits that could interfere with the understanding and execution of the task, such as, for instance, global aphasia, apraxia, dementia and depression. 4 patients and 8 controls reported previous experience in the use of computer games. The study followed accepted guidelines and was approved by the ethics committee of clinical research of the IMAS - Instituto Municipal de Asistencia Sanitaria (Barcelona, Spain).

**Table 1:** Patient Description

Group	ID	Age	Sex	Days after Stroke	Side of Lesion	Type of Stroke	**Barthel Index **[54]	**Brunnstrom Stage **[55]
Model Development	1	57	M	125	L	H	72	IV
	2	69	M	59	L	H	61	III
	3	57	M	120	L	I	100	VI
	4	43	F	21	R	I	96	V
	5	62	M	36	L	I	91	VI
	6	58	M	108	L	I	98	V
	7	73	M	135	L	I	84	IV
	8	45	M	24	L	H	56	V
	9	65	M	118	R	I	72	IV
	10	70	M	174	R	H	62	V
	11	58	M	176	L	H	78	V
	12	32	M	425	R	I	78	II

Descriptive		57.4	11/1	126.8	8/4	7/5	79.0	-
		(12.1)		(108.2)			(15.1)	

Model Assessment and Transfer Task	1	79	F	20	R	I	38	II
	2	60	F	6	R	H	42	III
	3	67	M	13	R	I	39	II
	4	55	M	15	R	I	41	II
	5	79	F	9	L	I	51	IV
	6	50	F	10	L	I	52	III
	7	52	M	20	R	H	31	II
	8	50	F	15	R	I	46	II
	9	69	M	10	R	I	43	III

Descriptive		62.3	4/5	13.1	2/7	7/2	42.6	-
		(11.7)		(4.9)			(6.5)	

The table shows sex with M = male and F = female, lesion side with L = left and R = right, and type of stroke with I = ischemic and H = hemorrhagic. The descriptive statistics show the mean and the standard deviation.

### Rehabilitation Gaming System (RGS)

**Figure 1:**
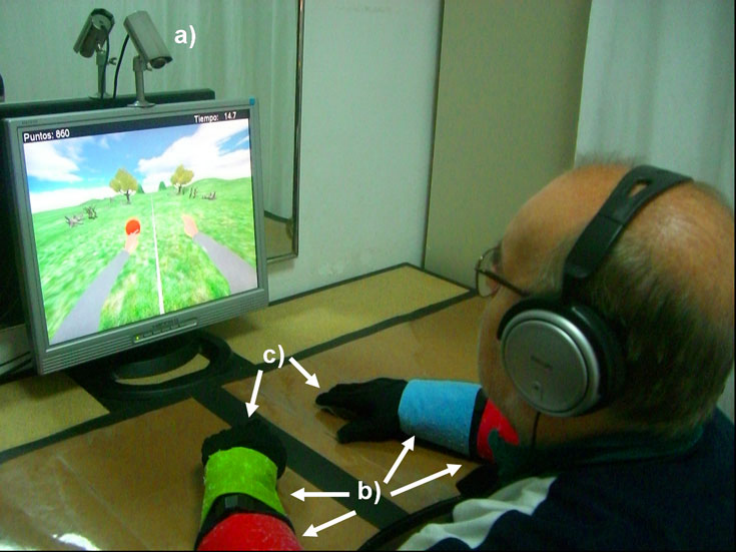
**The Rehabilitation Gaming System**. A subject sits on a chair with his/her arms on a table, facing a screen. Arm movements are tracked by the camera mounted on top of the display (a). The tracking system determines in real-time the position of the color patches positioned at wrists and elbows and maps these onto a biomechanical model of the upper extremities (b). Data gloves can be used to detect finger movements (c). On the display two virtual arms mimic the movements of the subject's arms.

The RGS is implemented using: a PC (Intel Core 2 Duo Processor, Palo Alto, USA) with graphics accelerator (nVidia GeForce Go 7300, Santa Clara, USA); a 17 inch LCD display (Samsung, Daegu, South Korea); a color CCD camera (KE-240CV, Camtronics, USA) positioned on top of the display (Figure 1a); four color patches (Figure 1b); and two 5DT data gloves (Fifth Dimension Technologies, Johannesburg, South Africa) (not used in the task described here) (Figure 1c). The virtual tasks are implemented with the Torque Game Engine (TGE, GarageGames, Oregon, USA). The movements of the upper extremities of the patient are tracked using the custom developed vision based motion capture system, AnTS [37] (see Additional File 1 for a detailed description).

### Virtual scenario

**Figure 2:**
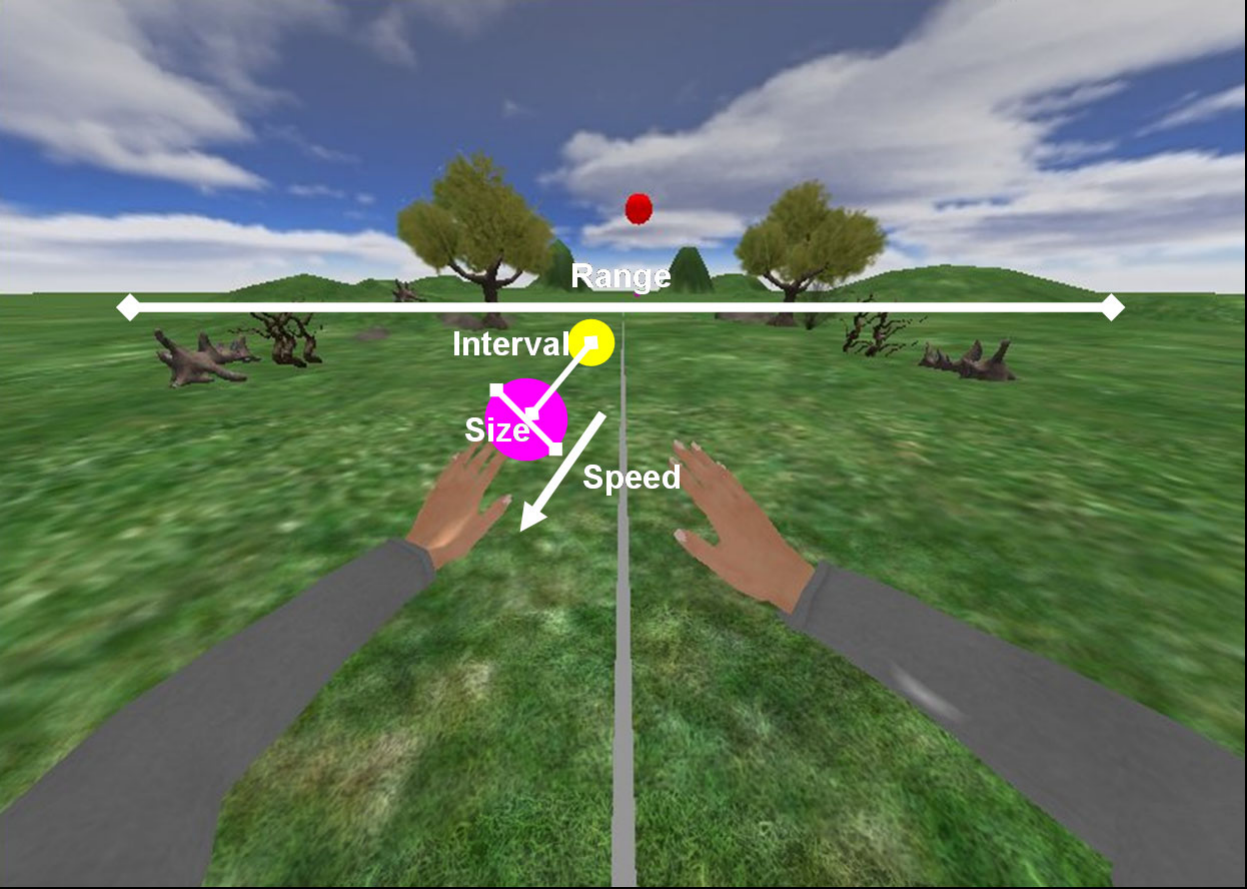
**Spheroids and the virtual environment**. The scenario represents a spring-like nature scenario. Within this scenario two virtual arms move accordingly to the movements of the user. The virtual arms are consistent with the orientation of the user, pointing towards the world, providing a first person perspective during the virtual interaction. The difficulty of the sphere interception task is modulated by the speed of the delivered spheres, the interval of appearance between consecutive spheres and the range of dispersion in the field of view. The gaming parameters are graphically described in the Figure.

The RGS scenario evaluated here, Spheroids, consists of a green landscape populated with a number of trees against the background of a mountain range. Integrated in the virtual world is a model of a human torso with arms positioned in such a way that the user has a first person view of the upper extremities (Figure 2). The movements of the user's physical arms that are captured by the motion capture system are mapped onto the movements of the virtual arms. The latter thus mimic the movements of the user.

In Spheroids, spheres move towards the user and these are to be intercepted through the movement of the virtual arms. Each time a sphere is intercepted, the user obtains a number of points that accumulate towards a final score. The task is defined by different gaming parameters, i.e. the speed of the moving spheres, the interval between the appearance of consecutive spheres and the horizontal range of dispersion of the spheres in the field of view (Figure 2).

### Calibration and diagnostics task

**Figure 3:**
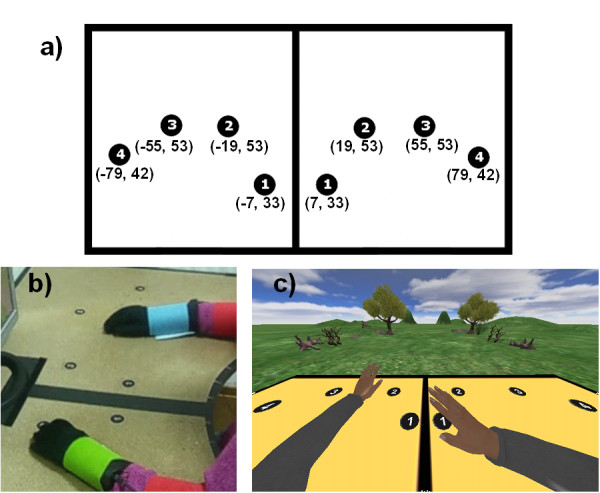
**Calibration task**. The user has to move his/her hands to numbered dots positioned on a tabletop. (a) Coordinates (in cm) of the target numbers to be reached. (b) Physical calibration task. The task is performed on the physical tabletop. (c) Virtual calibration task. Virtual replica of the physical calibration task. The instructions are the same as in the real task, but now the task is to be performed with the virtual arms on top of the virtual table.

In order to assess the ecological validity of the RGS task, we designed a directed pointing calibration and diagnostics task. This task evaluates specific properties of arm movements and analyzes their transfer between physical and virtual worlds. In this way RGS also obtains kinematics based diagnostic information. For the physical task, the user is asked to move his/her hands to numbered dots positioned at specific locations on the tabletop (Figure 3). There are four dots at each side of the table with increasing numbering corresponding to different reaching positions (Figure 3a). The user is instructed by a text displayed on the RGS screen and a pre-recorded audio statement to move one of the hands from a resting position to a new position indicated by a number corresponding to a position on the table top. In each trial every hand and target position is randomly defined by the system. The virtual version of the task is identical to the physical one and the user observes on the computer screen a virtual replica of the table top with the numbered dots and the task is to be performed this time in the virtual scenario (Figure 3c).

In both, its real and virtual version, the calibration task extracts information on the speed of movement, range of movement (combined shoulder and elbow aperture for arm extension) and latency (time to initiate a movement from a start cue). In the training sessions this information is used to compute the baseline parameters of Spheroids and thus the starting difficulty of the RGS training session. In addition, this calibration task is used to monitor the impact of training on arm kinematics over sessions. The calibration task always precedes every Spheroids session.

### Personalized Training Module

**Figure 4:**
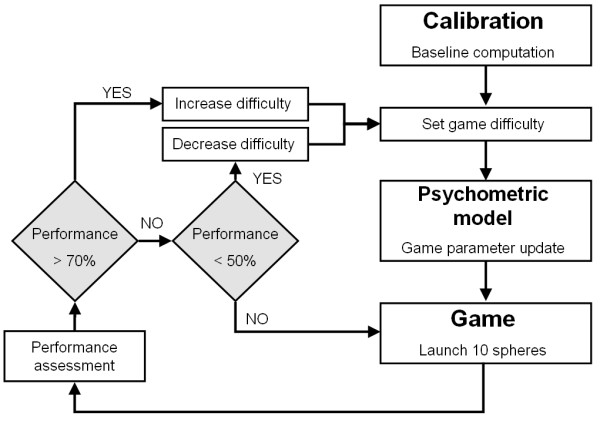
**Flow diagram of the RGS Personalized Training Module**. The game parameters are continuously updated based on the performance of the subject. This provides an automated adjustment of the difficulty of training over time based on a psychometrically validated user model.

The Personalized Training Module (PTM) can autonomously adjust the difficulty of the RGS sessions on a trial by trial basis. This automated procedure follows a number of steps (Figure 4). Before the training starts a baseline level is defined by means of the calibration task described above. After every block of ten trials, i.e. delivery of ten spheres, the PTM adjusts the difficulty level given the performance of the user. For each new difficulty value the corresponding gaming parameters are computed taking into account the previous response of the user to the individual parameters and the psychometric model of Spheroids.

In the instantiation of RGS presented here difficulty is increased with 10% when the user intercepts more than 70% of the spheres up to a maximum difficulty level of 100%. Conversely difficulty is lowered with 5% if the user intercepts less than 50% of the spheres. Hence, there is a continuous adaptation of the game parameters to the user's performance. Additionally, individualization is done for each arm separately, computing different difficulty levels and thus game parameters, for individual arms.

In the context of the PTM, the performance of an RGS user in the Spheroids task is assessed as a function of four individual parameters:

(1) Performance=f(Speed,Interval,Range,Size)

The investigation of the effect of these individual parameters on performance allowed us to establish a quantitative relationship between multiple independent input variables (game parameters) and a single output variable (difficulty). Considering the broader case of a non-linear relation between the input variables (task properties) and the performance of the subject, we used a quadratic model that takes into account first-order terms, interactions (cross-product terms) and second-order terms [38]. For three input variables (x_1_, x_2_, x_3_) and one output variable y this renders:

(2) $$\begin{array}{l} y=m_{0}+m_{1}\cdot x_{1}+m_{2}\cdot x_{2}+m_{3}\cdot x_{3}+\ldots \\ \ldots +m_{12}\cdot x_{1}\cdot x_{2}+m_{13}\cdot x_{1}\cdot x_{3}+m_{23}\cdot x_{2}\cdot x_{3}+\ldots \\ \ldots +m_{11}\cdot x_{1}^{2}+m_{22}\cdot x_{2}^{2}+m_{33}\cdot x_{3}^{2} \end{array}$$

where m_1_.x_1_...m_3_.x_3 _are the linear terms, m_12_.x_1_.x_2_...m_23_.x_2_.x_3 _are the interaction terms and m_11_.x_1_^2^...m_33_.x_3_^2 ^are the quadratic terms. By fitting the model to the data of interest, we can extract the regression parameters (m coefficients), which best describe the contribution of their respective terms or independent variables to the dependent variable. In our case we evaluated the m coefficients that relate the game parameters to task difficulty.

### Protocol

To be able to assess the relationship between game parameters and performance, stroke patients (n = 12) and controls (n = 10) performed Spheroids with random combinations of game parameters (i.e, speed, time interval, range and size). For a specific combination, each parameter could have one of four predefined values: Speed = [8, 14, 19, 25] m/s, Interval = [.25, .50, 1.0, 1.5] s, Range = [.42, .69, .83, .97] m, and Size = [.07, .14, .21, .28] m. We selected this set of parameters in order to cover the behaviorally relevant part of the parameter space while keeping the number of trials within practical limits. We varied the gaming parameters every 10 trials (i.e., 10 spheres) to cover the total number of 4^4 ^= 256 possible combinations. In each session, the user was exposed to a random subset of these combinations. To avoid fatigue, we did sessions of a maximum duration of 20 minutes. In a session of this duration the average number of combinations was 82 (~820 spheres). Although there could be repetition of combinations, we ensured that the full space of 256 possible combinations was covered for both, the patients and controls. Subsequently, for each combination of parameters we assessed the average success rate (number of successful sphere interceptions), separately for patients and controls. The data form controls allowed us to quantify the relation between performance and game parameters. The model was then fitted to the performance data from patients. Given the data generated in these trials we could extract the parameters of the psychometric model and define the PTM for the online adaptation of difficulty. To evaluate the performance of this psychometric model, two new groups of patients (n = 9) and controls (n = 10) performed a 20 min session of the automated Spheroids task. Additionally, to asses the transfer between the physical and virtual tasks in the RGS, the same group of patients (n = 9) and controls (n = 10) performed the physical and virtual versions of the calibration task.

### Usability

In order to assess the usability aspects of the RGS, the acceptance of the training and overall satisfaction concerning the use of RGS, the group of patients (n = 9) that performed the transfer task and the adaptive Spheroids session were given a 4-item self-report questionnaire. This questionnaire was presented in the format of a 5-point Likert scale and patients had to report their agreement/disagreement with respect to a number of statements. With this questionnaire we assessed a number of aspects such as enjoyment of the task, understanding and ease of the task, and subjective performance. Here we focused on the more general aspects related to the usability and acceptance of the RGS. Therefore, we reported on the answers given to two relevant questions of the questionnaire.

### Data analysis

To assess the main and interaction effects of the game parameters on the performance of the Spheroids task, we performed a four way analysis of variance (ANOVA) with the game score as the dependent variable and Speed, Interval, Range and Size as independent variables. Once we identified the main effects and interaction effects between the parameters of the training scenario and the user's performance, we quantified this relationship using a quadratic multiple regression model, and extracted the parameters of the regression for both patients and controls.

For the analysis of the performance data of the adaptive version of Spheroids, we extracted the difficulty level reached during the task (average of the 30 last trials) and the final score (percentage of successful sphere interceptions) separated for individual arms. Subsequently, to analyze the mismatch between the performance of the two arms, we computed the ratio of the difficulty between the paretic and the nonparetic arm in patients, and between nondominant and dominant arms for controls. The same analysis was done for the final score. A ratio of 100% would represent a perfect matching performance of the arms. We also analyzed the relation between the adapted gaming parameters for both groups of subjects, by computing the average of the individual parameters over the entire session.

For the analysis of transfer between physical and virtual environments, we extracted the average speed during movement and computed the speed ratio between arms. In addition, for both environments we analyzed the endpoint movement trajectories for successful arm extension movements between two points for both arms in patients and controls. Here, trajectories are considered those that successfully go between the two predefined fixed points - the same ones in both calibration tasks - with an endpoint precision error smaller than 10 cm.

Within-subject data were compared using a paired Student's t-tests or a Wilcoxon signed ranks test. For between-subject comparisons we used an independent sample t-test or a Mann-Whitney test. p-values were not corrected for multiple comparisons. The normality of the distribution was assessed using a single sample Lilliefors hypothesis test of composite normality. Average data is expressed as mean ± standard error of the mean in the text and the figures, unless otherwise stated. For all statistical comparisons the significance level was set to 5% (p < .05). All statistical analysis was performed using MATLAB 2008a (MathWorks Inc., Natick, MA, USA) and SPSS 16.0 (SPSS Inc., Chicago, IL, USA).

## Results

We first evaluated the basic properties of the RGS by a psychometric assessment of the performance of stroke patients and control subjects, leading to the development of the RGS' PTM. We additionally assessed the performance of patients and controls within the model. Finally, we showed how the performance of the users transfers between the physical and the virtual world.

### Psychometric model

**Figure 5:**
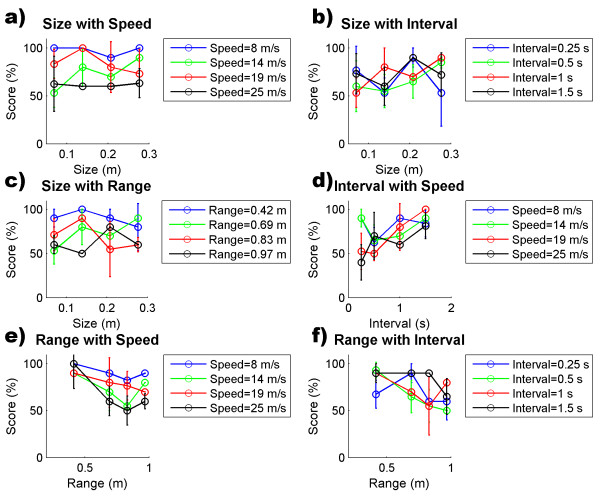
**Performance versus game parameters in control subjects**. a) Performance as a function of Size and Speed; b) Performance as a function of Size and Interval; c) Performance as a function of Size and Range; d) Performance as a function of Interval and Speed; e) Performance as a function of Range and Speed; f) Performance as a function of Range and Interval. Performance is measured as the percentage of successful sphere interceptions.

The Spheroids task is modulated by the Speed of the spheres, Interval of appearance between consecutive spheres, their Size, and Range of dispersal in the field (see Methods). The performance data of the controls showed that the size of the spheres had little effect, while Interval, Range and Speed substantially modulated performance (Figure 5). The 4-factor ANOVA revealed main effects of Speed (F(2.62) = 62.78, p < .001), Interval (F(2.62) = 64.41, p < .001) and Range (F(2.62) = 45.28, p < .001) while Size had no significant main effect (F(2.62) = 1.52, p = .2071). With respect to the interaction among the game parameters we observed that 3 of the 6 interactions had a significant effect: Speed*Interval (F(1.90) = 6.19, p < .001), Speed*Range (F(1.90) = 1.92, p = .0473) and Interval*Range (F(1.90) = 1.97, p = .0407). We did not find any further higher order interactions. Taking into account the significant effects, we can say that the difficulty of the task is defined by the Speed, Interval and Range, and by the interactions Speed*Interval, Speed*Range and Interval*Range, and this relation can be therefore quantified by a quadratic model (see Methods):

(3) $$\begin{array}{l} Difficulty=m_{0}+m_{1}\cdot Interval+m_{2}\cdot Speed+m_{3}\cdot Range+\ldots \\ \ldots +m_{4}\cdot Interval\cdot Speed+m_{5}\cdot Interval\cdot Range+m_{6}\cdot Speed\cdot Range+\ldots \\ \ldots +m_{7}\cdot Interval^{2}+m_{8}\cdot Speed^{2}+m_{9}\cdot Range^{2} \end{array}$$

where *Difficulty *is inversely proportional to the game's score. In this model, positive values of difficulty correspond to performance above average, while negative difficulty corresponds to performance below average.

For the controls we got a model fit (*R*^2 ^= 0.3745, *F(2.37) *= 82.4866, *p *< .001) with a Mean Squared Error (MSE) of 0.0463. In order to determine the generalization of the model, the stroke patients performed Spheroids following the same protocol. All patients were able to complete the task irrespective of their degree of impairment. Fitting our model to the data of the non-paretic hand we obtained a fit (*R*^2 ^= 0.3853, *F(2.37) *= 140.1967, *p *< .001) with a Mean Squared Error (MSE) of 0.0531 (see Additional File 2 for the fitting parameters). The goal of the psychometric model is to provide a single and "blind" adaptive rule for the update of the game parameters that can apply to all patients. Thus, the objective would be that the performance of the paretic arm equals that of the nonparetic one at the end of the treatment. For this reason we used the data of the nonparetic arm to fit the model because it represents an age matched approximation of the desired treatment outcome. We found that the correlation of the patients' model with the parameters of the fit of the healthy controls is .9557 (Pearson's correlation coefficient, p < .001). This means that the relationship between *Difficulty *and the parameters of Spheroids was consistent in both groups. Nevertheless, despite this correlation, the weights found for the patients are higher than for the controls. This can be explained by the fact that the same game parameters in both groups represent a more difficult task for the patients.

### Personalized Training Module

**Figure 6:**
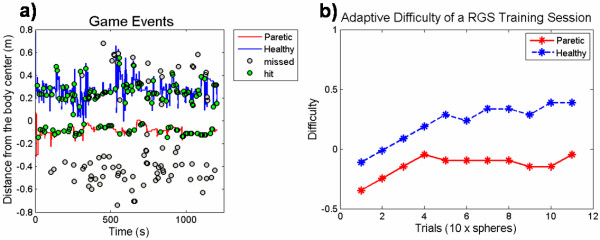
**Game events and task difficulty**. (a) Arm reaching distance over time for paretic (red) and healthy (blue) arms, and corresponding game events (hit and missed spheres). (b) Difficulty curves for paretic (red) and healthy (blue) arms over trials.

Given the fit of the data by the psychometric model we quantitatively defined the relationship between task difficulty and the game parameters allowing RGS to autonomously adjust the properties of the game to the abilities of the user with PTM. The automated procedure of PTM follows a number of defined steps (Figure 4). As an illustration of the application of the PTM, consider the performance and difficulty of the task achieved by a patient during a single training session separated for the paretic and non-paretic limbs (Figure 6). Analyzing the game events (Figure 6a), i.e. hit and missed spheres during the task, we observe a higher degree of failures on the paretic side because of a smaller range of movement. The detection of the successful and unsuccessful events for each arm was used by PTM to adjust the difficulty of the training specific to the performance of the considered arm. This means that we had an individual pattern of difficulty for each arm (Figure 6b).

**Figure 7:**
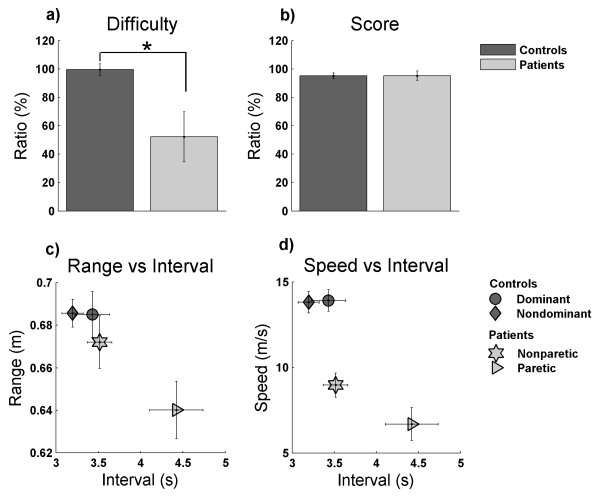
**Adaptive game results**. Difficulty (a) and score (b) ratios between the paretic and the nonparetic arms for patients (light grey); and between the nondominant and dominant arms for controls (dark grey). (c-d) Relation between game parameters for individual arms. * p < .05. Shown are means ± SEM.

The performance data from patients and controls in the PTM showed that the model captured the individual properties of the arms and adapted the difficulty level accordingly (Figure 7). As expected, the patients reached dissimilar difficulty levels for paretic and non paretic arms, as opposed to the case of the controls. Consequently, the difficulty ratio between arms was around 100% in controls (99.49 ± 4.11%) and lower in patients (52.27 ± 17.54%), and these were significantly different [t-test, t (8.8) = 2.62, p = .028] (Figure 7a). A correct adaptive procedure requires that the difficulty of the task is changed but the final score should be similar for both arms in controls and patients, and not different between groups. Indeed, the score ratio between arms in controls (95.17 ± 1.93%) and patients (95.21 ± 3.36%) was not significantly different [t-test, t (17) = -.009, p = .993] (Figure 7b).

We identified specific properties of the individual arms by exploring the individual gaming parameters (range, speed, and time interval between spheres) obtained for both arms in both groups, (Figure 7c, d). For control subjects, we found no significant differences between dominant and nondominant arms in *range *[t-test, t (9) = -.055, p = .957], *interval *[t (9) = 1.199, p = .261] and *speed *[t-test, t (9) = .233, p = .821]. This means that both arms showed similar properties during the task performance. On the other hand, for patients we found significant differences between paretic and nonparetic arms for *interval *[t-test, t (8) = -2.71, p = .027] and *speed *[z = -2.07, p = .038], the paretic arm being slower and requiring a longer time interval between consecutive spheres. The paretic arm also showed a smaller *range*, but the difference was not significant [Wilcoxon, z = -1.71, p = .086]. Comparing the performance of the individual arms between groups, the patients' paretic arm showed significantly lower *range *and *speed*, and a longer time *interval*, when compared with controls' dominant and nondominant arms (paretic-dominant: [t-test, t (17) = -2.64, p = .017] for *range*, [t-test, t (17) = 2.69, p = .015] for *interval *and (Mann-Whitney, z = -3.67, p = 2.2 × 10^-5^) for *speed*; paretic-nondominant: : [t-test, t (11.6) = -3.05, p = .010] for *range*, [t-test, t (10.5) = 3.61, p = .004] for *interval *and (Mann-Whitney, z = -3.59, p = 4.3 × 10^-5^) for *speed*). In contrast, patients' nonparetic arm showed a similar mean *interval *and *range *when compared to both arms of the controls (nonparetic-dominant: (Mann-Whitney, z = -1.06, p = .288) for *range *and [t-test, t (17) = .333, p = .743] for *interval*; nonparetic-nondominant: (Mann-Whitney, z = -.653, p = .514) for *range *and [t-test, t (17) = 1.66, p = .116] for *interval*). However, it had a significant lower *speed *(nonparetic-dominant: [t-test, t (17) = -5.26, p = 6.3 × 10^-5^], nonparetic-nondominant:[t-test, t (17) = -5.18, p = 7.6 × 10^-5^]).

In summary, the nonparetic arm of the patients showed similar properties as both arms of the control group, although being slower in the performance of the task. On the other hand, the paretic arm was noticeably different from the control group and also from the contralateral nonparetic arm. This means that our model was capable of capturing the specific features of the user for both arms and that it adapted the task parameters accordingly.

### Transfer between Real and Virtual Environments

**Figure 8:**
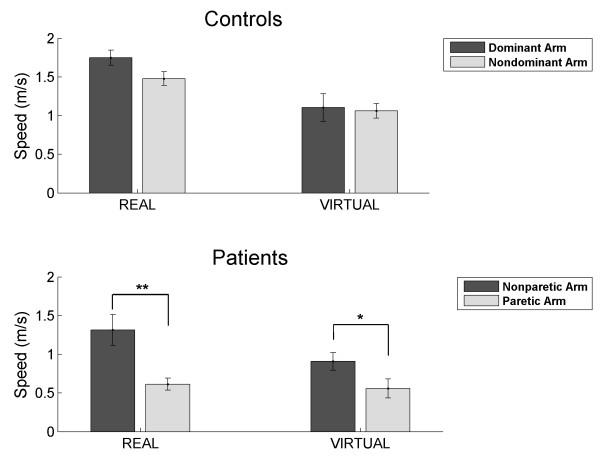
**Movement speed in an equivalent real and virtual calibration task**. Speed (mean ± SEM) for both arms, in controls and patients, in real and virtual environments. * p < .05, ** p < .01.

For the RGS training, it is essential to understand the transfer of performance between the virtual and the physical world. For the control subjects we observe a non-specific reduction in the speed of movement in the virtual world when compared to the real world ([t-test, t(8) = 4.324, p = .003] for the dominant arm and [t-test, t(8) = 2.992, p = .017] for the nondominant arm) (Figure 8 upper panel). This effect was not observed in the patient group ([t-test, t(8) = 1.896, p = .095] for the nonparetic arm arm and [t-test, t(8) = .453, p = .663] for the paretic arm). Nevertheless, for controls the relationship between arms was preserved in real and virtual worlds. Thus, the movement speed of the dominant and nondominant arms was not significantly different in both environments (real: [t-test, t (8) = 1.91, p = .093]; virtual: [t-test, t (8) = .296, p = .775]). For the stroke patients (Figure 8 lower panel) we observed that there was a significant difference between nonparetic and paretic arms in both real [t-test, t (8) = 4.565, p = .0018] and virtual [t-test, t (8) = 2.312, p = .049] environments. Specifically, the paretic-nonparetic speed ratio was 50.38 ± 6.14% in the physical task and 65.67 ± 17.75% in the virtual one, and these were not significantly different [Wilcoxon, z = -1.007, p = .314]. This means that although the specifics of the speed of movement were not transferred, the relationship between the speed of the arms was preserved and thus the deficit, understood as the relative speed difference between paretic and nonparetic arms, was consistently transferred between environments.

Comparing the speed of the individual arms between groups, we observed that the nonparetic arm of the patients was not significantly different from both arms of the control subjects in real and virtual worlds (nonparetic-dominant: [t-test, t (16) = -1.961, p = .068] for the real and [t-test, t (16) = -.925, p = .369] for the virtual task; nonparetic-nondominant: [t-test, t (16) = -.755, p = .461] for physical task and [t-test, t (16) = -1.040, p = .314] for virtual task). We observed that in all cases the speed of the paretic arm was significantly different from controls (paretic-dominant: [t-test, t (16) = -9.076, p = 1.1 × 10^-7^] for physical task and [t-test, t (16) = -2.508, p = .023] for virtual task; paretic-nondominant: [t-test, t (16) = -7.275, p = 1.8 × 10^-6^] for real task and [t-test, t (16) = -3.223, p = .006] for virtual task).

**Figure 9:**
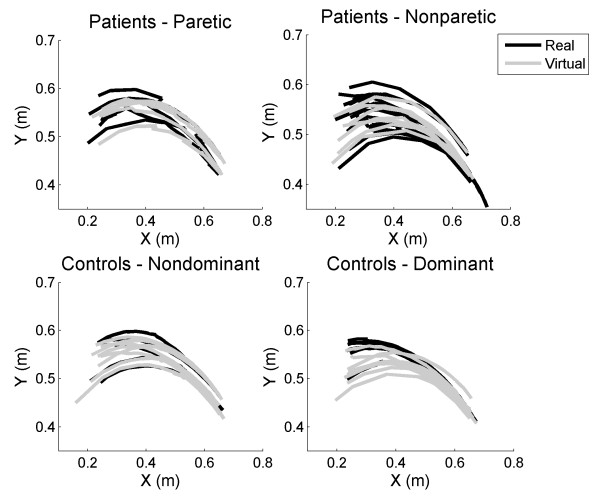
**Movement trajectories in an equivalent real and virtual calibration task**. Endpoint movement trajectories between two fixed points in the real and virtual calibration task for both arms, in controls and patients. Left arm movements are mirrored to the right side to allow for comparison.

Additionally, we examined the endpoint trajectories for successful arm extension movements. Extension movements between two fixed points in the real and virtual calibration tasks showed similar movement properties across environments (Figure 9). In general, patients showed more uneven movement patterns while controls showed smoother and more continuous movements. In the particular case of the patients, it can be clearly appreciated that less successful movements are achieved by the paretic arm, being the few successful ones more unstructured. On the other hand, controls showed a balanced control of both arms and reduced movement variability when compared to patients.

### Usability and Acceptance

In order to assess the acceptance of the RGS, patients were asked about the enjoyment and the ease of the task. To the statement "I enjoyed the task", 44.4% of the patients strongly agreed, 44.4% agreed and 11.1% neither agreed nor disagreed. To the statement "The task was easy", 22.2% strongly agreed, 55.6% agreed, 11.1% neither agreed nor disagreed and 11.1% disagreed. Based on these results and as an overall analysis we feel confident to conclude that the acceptance of the RGS and its tasks was very high.

## Discussion

Here we presented the Rehabilitation Gaming System, a novel paradigm for the rehabilitation of motor deficits after lesions to the central nervous system. RGS has a number of properties that are consistent with our current understanding of neuronal mechanisms of stroke and its aftermath, and the functional requirements of rehabilitative training. First, it is neuroscience based and exploits the neuronal processes of action observation and execution, learning and recovery and proposes corresponding rehabilitation strategies. Second, by virtue of using VR it allows for the flexible creation of scenarios directed towards specific needs. Third, the proposed task studied here follows an individualized training approach, adjusted to the capabilities of the user. And fourth, RGS measures quantitative performance data for continuous monitoring of the patient to evaluate his/her progress over time, complementing clinical standard evaluation. A key component of the RGS is the Personalized Training Module (PTM). We showed that it allows the automatic adjustment of the difficulty level of the task to the user. In addition, we showed that there is a consistent transfer of movement speed between physical and virtual tasks.

RGS exploits the observation of goal-oriented movements through a virtual representation of the body, allowing the training of specific components of movement through the systematic presentation of proprioceptive and visual feedback. It is widely accepted that feedback of one's actions in terms of movement patterns and movement outcomes is critical for motor learning [12,39,40]. In the specific case of the reinforced feedback provided by virtual environments, it has been observed that such environments optimize the learning of complex tasks in healthy subjects [41] and have a positive impact on recovery following stroke [17]. Such feedback could help enhancing the cortical changes associated with motor learning [42,43], facilitating the acquisition of new motor skills.

Other groups deployed VR systems for upper limb rehabilitation with different paradigms [16]. As examples we can find systems based on "learning by imitation" that make use of a virtual teacher, whose movements are to be followed by the user [44]; systems that provide reinforcing feedback concerning the quality of the movements and the goal of the tasks [45]; systems that provide haptic feedback [46]; systems that combine VR with robot-assisted training [47]; systems that use video capture virtual reality [48]; or even systems that use VR to support the generation of motor images for mental imagery based techniques [49]. However, RGS provides a new contribution to the field in the sense that it is unique in the integration of a number of explicit hypotheses on the neuronal substrate of perception, learning and recovery in a single platform, exploiting new insights in individualized task oriented training.

Of special relevance is the psychometric PTM of the RGS for online adaptation of task difficulty. This model was developed by analyzing the relation between performance and game parameters in stroke patients and controls. The individual game parameters are weighted to produce the appropriate game parameters that are adapted online to the individual capabilities of the user. One of the main points of this model is to ensure that the task remains constantly interesting and challenging, but without reaching high levels of demand that could result in frustration or anxiety [35]. Here we showed that with the PTM implemented in Spheroids we were able to capture specific features of both arms in patients and controls, and to adapt the difficulty of the task accordingly. In patients, we were able to identify a dissimilar pattern of performance and task parameters in paretic and nonparetic arms. The paretic arm always required a lower level of difficulty in order to sustain performance. Consequently, the difficulty ratio between arms was significantly lower than for controls, which showed a balanced performance for both arms. By analyzing the individual game parameters (speed of the spheres, time interval between consecutive arms and range of dispersion), the performance of the paretic arm of the patients was significantly different from the contralateral arm and from the control group. On the other hand, the nonparetic arm shared the same aspects of the game dynamics with both arms of the controls, except for speed, the nonparetic arm requiring a significantly slower sphere speed during the game. We think that this difference in the speed could be related to a general slowing down in movements that has been reported in stroke patients [50,51].

In order to ensure the ecological validity of training with RGS we showed that aspects of the movement kinematics, such as endpoint trajectories and deficit properties, were transferred between two equivalent tasks in real and virtual environments. Consistent with previous research on movement transfer in physical and virtual environments we observe that healthy subjects were slower in the virtual environment [52], an effect not displayed by the patient group.

Here we propose RGS as a generic paradigm for neurorehabilitation that is currently applied specifically to motor deficits of the upper extremities. However, we believe that this concept will smoothly generalize to address other deficits of the skeletal-motor system resulting from CNS lesions and possibly more central perceptual and cognitive deficits such as neglect. We are currently developing new training protocols for rehabilitation in clinics and for continuous long-term at home diagnostics and training that will allow us to directly validate these assumptions.

We believe that the amalgamation of relevant features of the RGS makes it a valuable rehabilitation tool. The impact of RGS is currently the focus of studies with both acute and chronic patients and preliminary results support this belief [21,53].

## Conclusions

The Rehabilitation Gaming System uses Virtual Reality technology to implement training protocols in order to provide neurorehabilitation training that allows for a gradual and individualized treatment of deficits of the upper extremities after stroke. In the near future we will evaluate the use of RGS in longitudinal clinical studies with stroke patients in both acute and chronic stages. We expect to assess its specific impact on recovery and in the management of daily life.

## Competing interests

The authors declare that they have no competing interests.

## Patient's consent

Written informed consent was obtained from the patient for publication of this case report and accompanying images. A copy of the written consent is available for review by the Editor-in-Chief of this journal.

## Authors' contributions

MSC, SBB and PFMJV participated in the concept and development of the Rehabilitation Gaming System. MSC and EDO were main contributors in the acquisition of the data. MSC, SBB and PFMJV analysed and interpreted the data. All authors revised and approved the current version of the manuscript.

## Supplementary Material

Additional file 1 **Tracking System - AnTS**. Description and operation of the vision based tracking system AnTS. Here we describe how AnTS tracks colored patches placed at the wrists and elbows of the user, to map the movements of the user onto the movements of the avatar. Click here for file

Additional file 2 **Performance versus Gaming Parameters**. Identification of the main effects and interaction effects between the parameters of the training scenario and the user's performance through a four way analysis of variance (ANOVA) with the game score as the dependent variable and Speed, Interval, Range and Size as independent variables. Here we show the quantification of this relationship, through the extraction of the parameters of the quadratic multiple regression for both patients and controls. Click here for file
