# Supplementary material for: Neurorehabilitation using the virtual reality based Rehabilitation Gaming System: methodology, design, psychometrics, usability and validation
Source: J Neuroeng Rehabil. 2010 Sep 22;7:48. doi: 10.1186/1743-0003-7-48 (PMC2949710; doi:10.1186/1743-0003-7-48)
Supplement: Additional file 1 — Tracking System - AnTS. Description and operation of the vision based tracking system AnTS. Here we describe how AnTS tracks colored patches placed at the wrists and elbows of the user, to map the movements of the user onto the movements of the avatar. [file 1743-0003-7-48-S1.DOC]

**ADDITIONAL FILE 1**

**Tracking System – AnTS**

The movements of the upper extremities of the RGS users were tracked using the custom vision based tracking system AnTS. AnTS is a general purpose multiple object tracking tool based on Bayesian inference that contains a number of filters and color tracking methods as well as lens distortion and perspective correction techniques [1]. In RGS AnTS tracks unique colored patches placed at the wrists and elbows of the user. In this way the visual segmentation task is easily resolved and potential ambiguities due to the crossing of the upper extremities are avoided. AnTS maps the RGB values received from the 640x480 image onto a Hue Saturation Value (HSV) color space. In this color space, the hue value alone encodes for the color identity of the markers, which makes their tracking more robust to changes in light conditions. AnTS uses Bayesian probabilistic methods to infer the most likely position of each of the patches given the image stream. This method is used to solve occlusions and crossing related problems given the known properties of the different color patches (size, color, movement history, etc). Once the color patches are located, a bio-mechanical model of the human torso is used to compute the joint angles for shoulder and elbow of both arms. In the design of the RGS tracking system we purposefully imposed the use of only one camera as a constraint. As a consequence, the system calibration requirements are reduced as well as its computational requirements. In order to map the tracked markers captured with a single camera to the 8 joint angles (pitch and yaw of the 4 tracked joints) of the avatar we use a perspective correction to reduce distortions due to optics and view angle and a model based alignment based on the human skeleton. The latter constraint prevents the motion capture system to deliver unrealistic joint angles. Hence, a number of approximations have been made to recreate 3D movements from a single 2D image (Add_Figure 1).


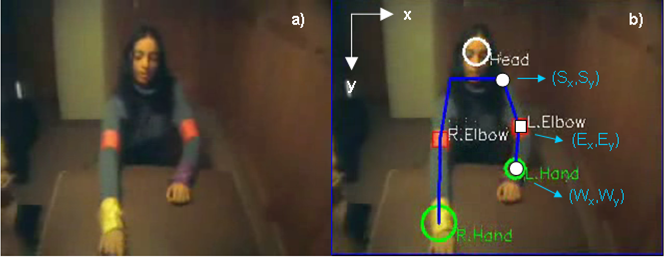


Add_Figure 1. Joint angle detection performed by the AnTS tracking system. a) Original image acquired by the RGS color camera. A subject is wearing color patches at wrists and elbows. b) AnTS color patch detection with a simplified upper-body model superimposed. The axis as well as the coordinates of the relevant points used to compute the left arm joint angles are indicated.

The computation of the joint angles always starts with the localization of the head, left and right elbows (L.Elbow, R.Elbow), and left and right wrists (L.Wrist, R.Wrist) according to its colors (Add_Figure 1).

Taking as an example the left arm, the estimated direction vectors of the shoulder-elbow segment, *SE*, are:

(1)

where β is the angle of attack of the video camera. Accordingly, the estimated pitch and yaw angles of the shoulder joint are given by:

(2)

where ║SE║=0.71d, where d defines the average shoulder width.

Using the same reasoning, the estimated direction vectors of the elbow-wrist segment, *EW*, are:

(3)

where β is the angle of attack of the video camera and the estimated pitch and yaw angles of the elbow joint are given by:

(4)

where ║EW║=0.59d.

The motion capture system runs at an update rate of 30 Hz and the median error in the reconstruction of the angles is 11 degrees.

**References**

[1] Mathews Z, Bermúdez i Badia S, Verschure PFMJ. A Novel Brain-Based Approach for Multi-Modal Multi-Target Tracking in a Mixed Reality Space. *INTUITION - International Conference and Workshop on Virtual Reality 2007*. Athens, Greece 2007.
