# Supplementary material for: Neurorehabilitation using the virtual reality based Rehabilitation Gaming System: methodology, design, psychometrics, usability and validation
Source: J Neuroeng Rehabil. 2010 Sep 22;7:48. doi: 10.1186/1743-0003-7-48 (PMC2949710; doi:10.1186/1743-0003-7-48)
Supplement: Additional file 2 — Performance versus Gaming Parameters. Identification of the main effects and interaction effects between the parameters of the training scenario and the user's performance through a four way analysis of variance (ANOVA) with the game score as the dependent variable and Speed, Interval, Range and Size as independent variables. Here we show the quantification of this relationship, through the extraction of the parameters of the quadratic multiple regression for both patients and controls. [file 1743-0003-7-48-S2.DOC]

**ADDITIONAL FILE 2**

**Performance versus Gaming Parameters**

In order to quantify the behavioral data we performed a four way analysis of variance (ANOVA) with the game score as the dependent variable and Speed, Interval, Range and Size as independent variables.

The ANOVA analysis reveals a significant main effect of speed (F(2.62)=62.78, p<0.001), interval (F(2.62)=64.41, p<0.001) and range (F(2.62)=45.28, p<0.001). The size has no significant main effect (F(2.62)=1.52, p=0.2071). On what concerns interaction effects, there is significance in some of the second order interactions, namely Speed*Interval (F(1.90)=6.19, p<0.001), Speed*Range (F(1.90)=1.92, p=0.0473) and Interval*Range (F(1.90)=1.97, p=0.0407). Third and fourth order interactions do not reach significance.

To identify the exact relationship between the parameters of the training scenario and the user’s performance we quantify this relationship using a quadratic multiple regression model:

(1)

where *Difficulty* is inversely proportional to the game’s score. For the healthy controls we get a model fit (*R*^2^ = 0.3745, *F(2.37)* = 82.4866, *p* <.001) with:

(2)

and a Mean Squared Error (MSE) = 0.0463.

Fitting our model to the data of the non-paretic hand of the acute stroke patients (*R*^2^ = 0.3853, *F(2.37)* = 140.1967, *p* <. 001) we obtained:

(3)

with a Mean Squared Error (MSE) = 0.0531.
